# The Effect of Cyanobacterial Biomass Enrichment by Centrifugation and GF/C Filtration on Subsequent Microcystin Measurement

**DOI:** 10.3390/toxins7030821

**Published:** 2015-03-10

**Authors:** Shelley Rogers, Jonathan Puddick, Susanna A. Wood, Daniel R. Dietrich, David P. Hamilton, Michele R. Prinsep

**Affiliations:** 1 Department of Chemistry, School of Science, University of Waikato, Private Bag 3105, Hamilton 3240, New Zealand; E-Mails: sr110@students.waikato.ac.nz (S.R.); m.prinsep@waikato.ac.nz (M.R.P.); 2 Environmental Research Institute, University of Waikato, Private Bag 3105, Hamilton 3240, New Zealand; E-Mails: susie.wood@cawthron.org.nz (S.A.W.); d.hamilton@waikato.ac.nz (D.P.H.); 3 Cawthron Institute, Private Bag 2, Nelson 7010, New Zealand; 4 Human and Environmental Toxicology, University of Konstanz, P.O. Box 662, 78457 Konstanz, Germany; E-Mail: daniel.dietrich@uni-konstanz.de

**Keywords:** microcystin, cyanobacteria, *microcystis*, *planktothrix*, biomass concentration, sample processing

## Abstract

Microcystins are cyclic peptides produced by multiple cyanobacterial genera. After accumulation in the liver of animals they inhibit eukaryotic serine/threonine protein phosphatases, causing liver disease or death. Accurate detection/quantification of microcystins is essential to ensure safe water resources and to enable research on this toxin. Previous methodological comparisons have focused on detection and extraction techniques, but have not investigated the commonly used biomass enrichment steps. These enrichment steps could modulate toxin production as recent studies have demonstrated that high cyanobacterial cell densities cause increased microcystin levels. In this study, three microcystin-producing strains were processed using no cell enrichment steps (by direct freezing at three temperatures) and with biomass enrichment (by centrifugation or GF/C filtration). After extraction, microcystins were analyzed using liquid chromatography-tandem mass spectrometry. All processing methods tested, except GF/C filtration, resulted in comparable microcystin quotas for all strains. The low yields observed for the filtration samples were caused by adsorption of arginine-containing microcystins to the GF/C filters. Whilst biomass enrichment did not affect microcystin metabolism over the time-frame of normal sample processing, problems associated with GF/C filtration were identified. The most widely applicable processing method was direct freezing of samples as it could be utilized in both field and laboratory environments.

## 1. Introduction

Microcystins (MCs) are hepatotoxic compounds produced by freshwater, marine and terrestrial cyanobacteria globally. They pose a significant risk to human and animal health if contaminated water or food products are ingested [1]. Microcystins accumulate in the livers of animals where they irreversibly inhibit serine/threonine phosphatase enzymes, inducing hepatotoxicity [2]. In addition, chronic exposure to microcystins has been linked to liver tumor promotion [3,4,5] and MC-LR has been categorized by the World Health Organization as “probably carcinogenic for humans” [6].

Currently, microcystin concentrations in environmental samples are determined using protein phosphatase inhibition assays, enzyme-linked immunosorbent assay (ELISA), chemical derivatization with gas chromatography-mass spectrometry analysis, and high performance liquid chromatography (HPLC) coupled to either ultra-violet or mass spectrometry detection [7,8]. Due to a broad range in sensitivity, selectivity and consumable/equipment costs, each of these analytical methods is still used in laboratories across the world.

When cyanobacterial samples for microcystin analysis are collected in the laboratory or field, the biomass is generally concentrated prior to preservation or extraction. Samples collected in the field may also be transported for hours or days before preservation. Biomass concentration is usually accomplished by centrifugation or filtration on to glass-fiber filters (e.g., GF/C) [9]. These concentrated samples are routinely preserved by freezing at low temperature (≤−20 °C) and/or lyophilization. Different extraction/cell disruption methods are used and may involve use of solvents such as methanol or butanol [10], freezing and thawing [11], sonication [12,13], or microwave-enhanced extraction [14]. Most methodological studies have focused on comparing different microcystin extraction methods [9,14,15,16,17,18], but have not specifically investigated the effect of biomass enrichment prior to preservation and/or extraction.

The rationale for investigating the effect of biomass enrichment are several field-, mesocosm- and laboratory-based studies which demonstrated that high cyanobacterial cell densities can cause an increase in the production of microcystins and other cyanobacterial peptides [19,20,21]. Therefore processes used to enrich cyanobacterial biomass during sample preparation could affect microcystin metabolism and change the measured microcystin concentration. In this study, we compared several commonly used methods of processing cyanobacterial samples for subsequent microcystin analysis. The aim was to determine whether biomass enrichment steps altered microcystin metabolism and increased the measured cell quota (the amount of toxin per cell).

## 2. Results and Discussion

### 2.1. The Effect of Cyanobacterial Biomass Enrichment through Sample Processing Procedures

The microcystin concentrations of a colonial *Microcystis* strain (CAWBG11) [22], a single-celled *Microcystis* strain (CAWBG16) [23] and a filamentous *Planktothrix* strain (CAWBG59) [24] were quantified using liquid chromatography with tandem mass spectrometry detection (LC-MS/MS). Each strain was sampled using the following methods; direct freezing with no prior cell concentration (at three temperatures), centrifugation followed by lyophilization, or filtration on to GF/C filters followed by lyophilization.

**Figure 1:**
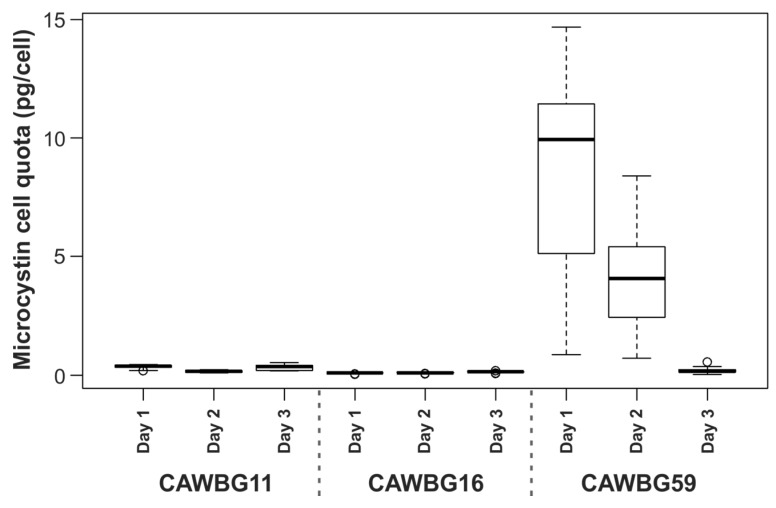
Microcystin cell quota across all treatments for each strain on different experimental days (solid black line shows median, box shows 1st and 3rd quartiles, whiskers extend to the last data point within 1.5-times the interquartile range, open circles are outliers beyond this range).

The microcystin cell quota varied significantly between cyanobacterial strains and between sampling days for each strain (Figure 1). This was particularly pronounced for the *Planktothrix* strain (CAWBG59). To compensate for this, data analysis was conducted on microcystin quotas normalized against a control sample collected for each strain on each day (subsamples frozen in liquid nitrogen, lyophilized and extracted in methanol).

**Figure 2:**
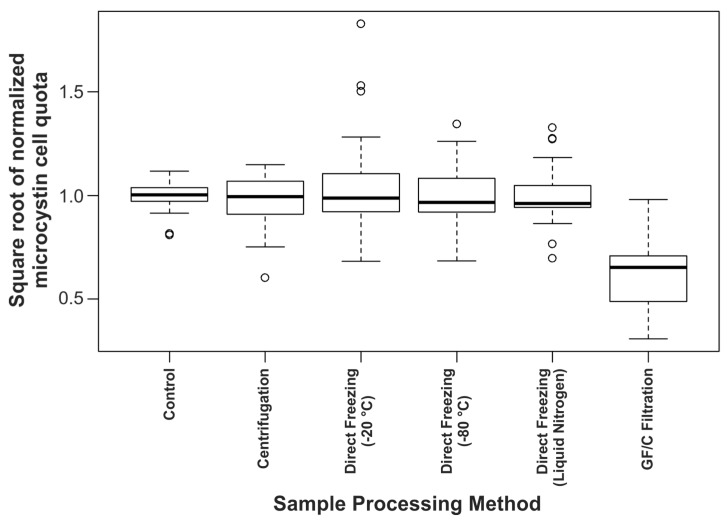
Comparison of the different sample processing methods, where microcystin cell quotas were normalized to the corresponding control sample to compensate for inter-strain and inter-day variability (solid black line shows median, box shows 1st and 3rd quartiles, whiskers extend to the last data point within 1.5-times the interquartile range, open circles are outliers beyond this range).

Comparison of the normalized microcystin quota for each sampling method showed consistent results for five of the six techniques (Figure 2). There was no significant difference in microcystin quota between the three direct freezing temperatures (−20 °C, −80 °C and liquid nitrogen) or when centrifugation was used to enrich cells. This indicates that the speed at which samples froze did not affect microcystin metabolism, and that a rapid enrichment of biomass during sample preparation does not influence microcystin quotas. The filtration method had a significantly lower microcystin yield than all other treatments (*p* < 0.001; Figure 2). The premise for this investigation was the association between increased microcystin production and increased cell density, based on several recent studies [19,20,21]. As GF/C filtration resulted in reduced microcystin concentrations, the observed effect was unlikely to be from cellular metabolism as high cell density has been shown to cause increased microcystin levels [19].

### 2.2. Investigation of the Low Microcystin Yields from GF/C Filtration Samples

Several processes could have led to the lower microcystin concentrations observed in the GF/C filtration samples; whole cells could have passed through the GF/C filter, cells could have lysed during the filtration process allowing toxin through the filter, microcystins could have adhered to the GF/C filters and/or cells could have become embedded in the filters, impeding cell lysis and reducing extraction efficiency. To investigate whether whole cells had passed through the GF/C filter, further samples were filtered by GF/C filtration only, and GF/C filtration followed by 0.2-µm filtration. The resulting filtrates were subjected to four freeze-thaw cycles (in order to lyse cells that may have passed through the filters) and were analyzed by LC‑MS/MS. There was no significant difference in the microcystin concentration of the filtrate samples (*p* = 0.11) indicating that whole cells were not passing through the GF/C filter. Additionally, during the study, there were no observed differences in the microcystin concentrations of the centrifugation supernatants and the GF/C filtrates (*p* = 0.19), indicating that cell lysis during the filtration process was not the cause.

**Figure 3:**
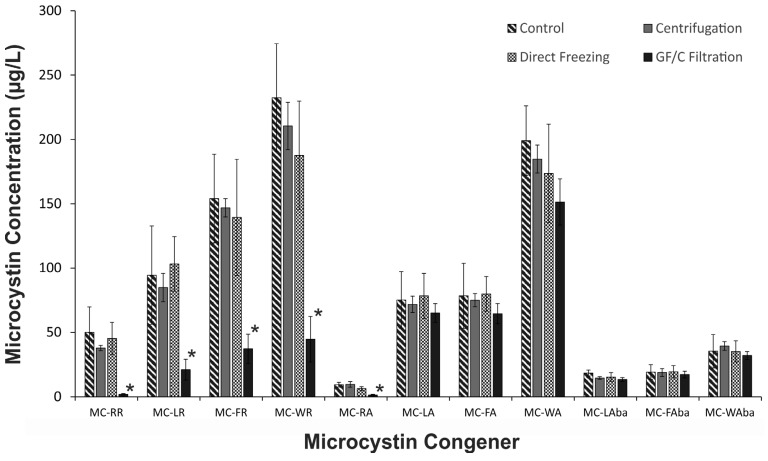
Microcystin concentration for selected microcystin congeners from *Microcystis* CAWBG11, where samples have been processed by different enrichment/preservation techniques (direct freezing samples using liquid nitrogen are displayed; only samples from Day 1 are presented; *n* = 3; error bars indicate ±1 standard deviation; * *p* < 0.05 compared to control, centrifugation and direct freezing samples).

When the individual microcystin congeners from *Microcystis* CAWBG11 were assessed, we observed that the low yields from the GF/C filtration samples were restricted to the arginine-containing microcystin congeners (e.g., MC-RR, MC-LR, MC-FR, MC-WR, MC-RA, *etc.*; Figure 3). All microcystins which contained arginine were present at significantly lower concentrations in the GF/C filtration samples compared to the other sampling methods (*p* ≤ 0.016). The concentrations of the hydrophobic microcystins (e.g., MC-LA, MC-FA, MC-WA, MC-LAba, MC-FAba, MC-WAba, *etc.*) were not significantly different between sampling methods (*p* ≥ 0.11). As *Microcystis* CAWBG16 and *Planktothrix* CAWBG59 produce only arginine-containing microcystins, the congener-dependent microcystin yield was not evident in these cyanobacterial strains.

**Figure 4:**
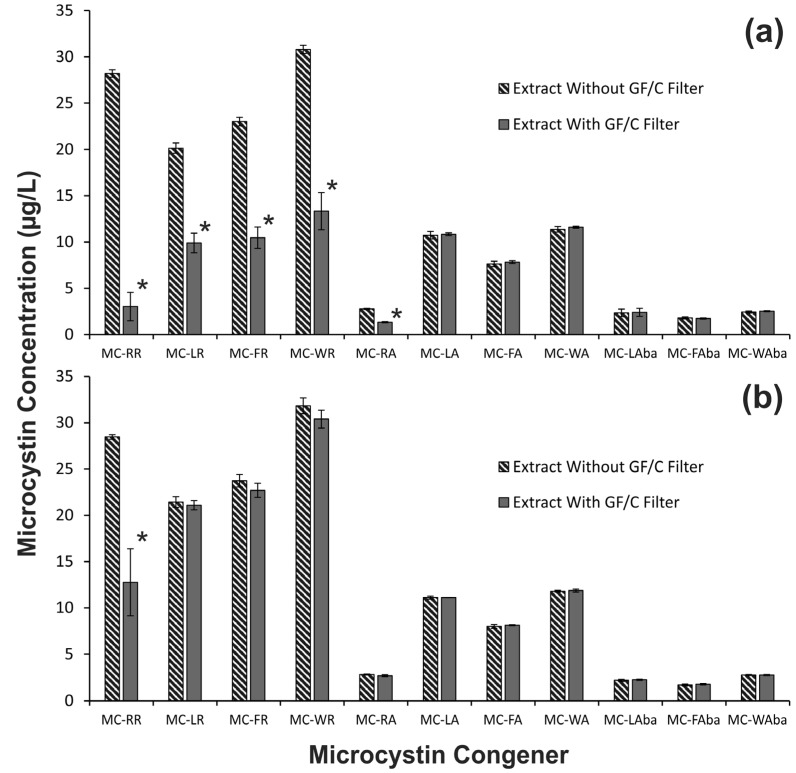
Selective adsorption of arginine-containing microcystin congeners to GF/C filters was assessed using (**a**) a methanol extract of *Microcystis* CAWBG11 and (**b**) a methanol extract of *Microcystis* CAWBG11containing 0.1% formic acid, which were incubated with or without GF/C filters (*n* = 3; error bars indicate ±1 standard deviation; * *p* < 0.001 compared to control).

The observed decrease in the yield of arginine-containing microcystins could be due to inefficient extraction or adsorption of the congeners to the GF/C filters. To test if filter adsorption was the cause, a GF/C filter was added to methanol extracts of CAWBG11 with and without formic acid. There were significantly lower concentrations of the arginine-containing microcystins when the GF/C filter was added to the methanol extract without formic acid (*p* < 0.001; Figure 4a), whilst concentrations of the hydrophobic microcystin congeners were not affected. When 0.1% formic acid was present, the adherence effect was reduced for the congeners containing a single arginine (Figure 4b); however, the concentration of MC-RR (which contains two arginine residues) was still reduced (*p* < 0.001).

The concentration of the hydrophobic microcystin congeners found in *Microcystis* CAWBG11 were comparable between the GF/C filtration samples and the samples processed by direct freezing and centrifugation (Figure 3). Therefore, it is unlikely that the filters were impeding cell lysis and reducing extraction efficiency. This suggests the cause for the low microcystin yield observed in the filtration samples was due to adsorption of arginine-containing microcystins to the GF/C filters during the extraction process.

### 2.3. Temporal Effect of Sample Centrifugation on Microcystin Metabolism

**Figure 5:**
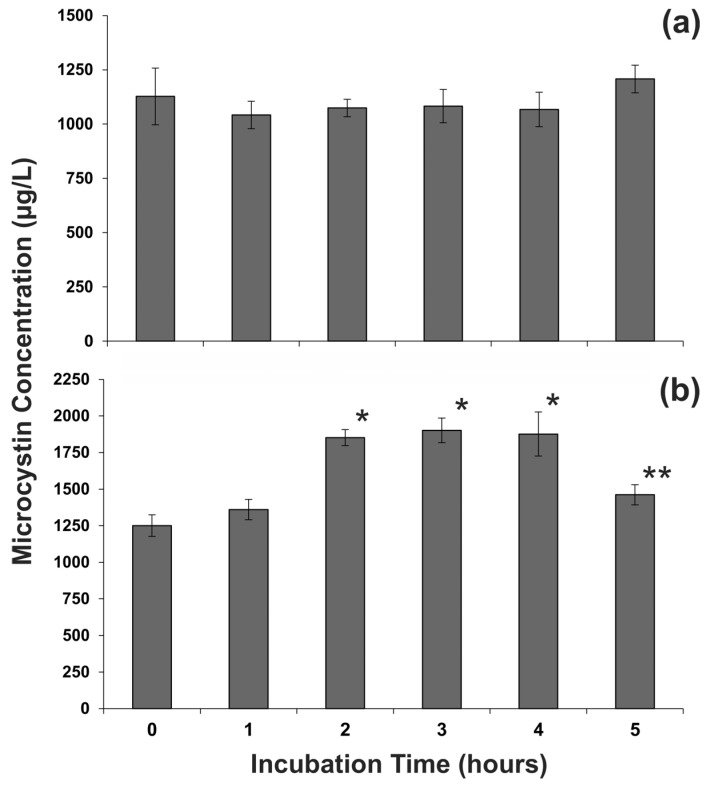
Cellular microcystin concentration of centrifuged *Microcystis* CAWBG16 which was incubated at ambient temperature (**a**) without removing the supernatant and (**b**) with the supernatant removed (*n* = 3; error bars indicate ±1 standard deviation; * *p* < 0.001 compared to 0 and 1 h samples; ** *p* < 0.05 compared to 2, 3 and 4 h samples).

To investigate whether a metabolic effect from centrifugation would occur after a longer incubation period, samples of *Microcystis* CAWBG16 were centrifuged and left at ambient temperature for up to 5 h. The samples were either incubated with or without the supernatant. During the 5 h time-course, there was no difference in microcystin concentration for the cell pellets covered with supernatant (*p* = 0.22; Figure 5a). However, when the supernatant had been removed and the cell pellet was left exposed to air, a 50% increase in microcystin concentration was observed after 2 h (*p* < 0.001; Figure 3b). The microcystin concentration remained high for an additional 2 h before decreasing at 5 h (*p* < 0.05; Figure 5b).

These data indicate that cyanobacterial samples concentrated by centrifugation could be left covered with the supernatant for up to 5 h prior to preservation, although care should be taken when moving the samples to ensure that the cells do not resuspend. However, once the supernatant was removed, there was a limited period of time (1 h) before a change in microcystin concentration was observed. The increase in microcystin concentration was likely due to changes in microcystin metabolism, but whether this was through increased production [20] or liberation of bound microcystin [25,26,27] is not clear. The cause for the altered metabolism could have been direct contact with air (possibly resulting in increased oxidative stress [27]), which may have affected the physiological functioning of the exposed cells. Further research would be required to confirm this and might involve laboratory or field studies where cyanobacteria are exposed to oxygen for different lengths of time. The decrease in microcystin concentrations after 4 h was a surprising observation and the mechanism for the reduction in microcystin concentration is not clear. It could be mediated by catabolism of the microcystin or sequestration of microcystins on to cellular components such as proteins or the thylakoid [25,26,27]. The entire sample was extracted and analyzed; therefore, microcystin export could not account for the observed decrease. Regardless of the underlying biochemical mechanism, the results indicate that centrifuged samples should be preserved promptly once the supernatant has been removed.

### 2.4. Sample Processing Considerations

Apart from GF/C filtration, the different sample processing methods produced comparable microcystin quotas for the three cyanobacterial strains assessed (Figure 2), indicating that biomass enrichment by centrifugation does not increase microcystin metabolism over normal sample processing time-frames. The lower microcystin quotas observed for the samples processed by filtration were due to arginine-containing microcystins binding to the GF/C filters (Figure 4). However, it appears that biomass enrichment by GF/C filtration did not cause an increase in microcystin metabolism as there was no increase in the concentration of hydrophobic congeners in these samples (Figure 3).

As cyanobacterial biomass enrichment did not increase microcystin metabolism (under normal sample processing time-frames), the decision regarding which method to utilize is dependent on the available equipment, time constraints and ease of processing. For laboratory-based experiments, biomass enrichment by centrifugation would be a convenient method. However, many planktonic cyanobacteria, including *Microcystis*, contain gas vesicles that assist in buoyancy [28]. Even after lengthy centrifugation, these cells can remain suspended, particularly when working with colonial *Microcystis* [29]. Bursting gas vesicles with increased pressure prior to centrifugation can alleviate this [30], although this was not tested in the current study and could result in cell lysis and release of microcystin into the supernatant. If centrifugation is used for biomass enrichment prior to microcystin extraction, researchers should preserve the samples shortly after removing the supernatant as microcystin metabolism is upregulated within 2 h (Figure 5b).

Centrifugation is less applicable in the field and preserving samples directly in liquid nitrogen would be more appropriate, although a separate filtered sample must be collected to determine the extracellular microcystin concentration. The cellular fraction can then be calculated by subtracting the extracellular microcystin concentration from the total microcystin concentration. Our study also showed that directly freezing cyanobacterial samples at −20 °C and −80 °C provided microcystin quotas comparable to those obtained via snap-freezing in liquid nitrogen. In a laboratory setting, direct freezing at these temperatures would likely be more convenient than freezing in liquid nitrogen.

The adsorption of microcystin congeners to GF/C filters is of concern as filtration is commonly used when collecting environmental samples for microcystin analysis [31]. Our data showed that arginine-containing microcystins adhered to GF/C filters (Figure 4a), but this effect could be partially alleviated through the addition of formic acid to the extraction solvent (Figure 4b). Other researchers have previously shown adsorption of microcystins to plastics and glass [32,33,34], but interestingly, this was shown to be more prevalent with hydrophobic microcystins than arginine-containing microcystins [33]. 

Binding of microcystins to plastics and glass have been noted when microcystins were dissolved in an aqueous solution [33,34]. We did not observe this adsorption effect with the aqueous extraction conducted on the direct freezing samples assessed during this study (Figure 2). However, we would still advise that control samples are included (such as the methanol extracted samples used in this study).

To improve understanding of the variables that regulate microcystin production and their ecological function, there is a need for greater standardization of procedures so that comparisons can be made between data sets. Previous studies have used a wide range of analytical methodologies (e.g., ELISA, LC-MS/MS) with the results normalized to a variety of parameters such as dry weight, cell number and optical density [1]. Whilst we have not proposed a universal method for processing microcystin samples, the present study has shown that samples preserved by direct freezing and samples enriched by centrifugation yield comparable results. We also identified that arginine-containing microcystins adhere to GF/C filters. Whilst this was less pronounced when acidified methanol was used as an extraction solvent, MC-RR congeners still adhered to the filters in the presence of 0.1% formic acid. We suggest that caution and careful method validation is undertaken when GF/C filtration is required.

## 3. Experimental Section

### 3.1. Cyanobacterial Strains

A colonial *Microcystis* strain (CAWBG11) [22], a single-celled *Microcystis* strain (CAWBG16) [23] and a filamentous *Planktothrix* strain (CAWBG59) [24] were used for this study (Cawthron Institute Microalgae Culture Collection, Nelson, New Zealand). The cultures were grown in MLA medium [35] at 20 °C under a 12 h:12 h light/dark cycle (photon-flux of 100 μmol·m^−2^·s^−1^; Biosyn 6000 CP Incubator (Contherm, Upper Hutt, Wellington, New Zealand) in either screw-capped plastic containers (60 mL) or glass Erlenmeyer flasks (500 mL).

### 3.2. Comparison of Sample Processing Methods

Three replicate experiments were carried out on different days for each strain. The samples were processed by: (i) direct freezing (no prior cell concentration) at −20 °C, −80 °C and in liquid nitrogen (−196 °C); (ii) cell concentration by centrifugation; or (iii) cell concentration by GF/C filtration. Triplicate subsamples (1 mL) for each treatment were taken from the same batch of culture after swirling to ensure homogeneity and processed as described below. Triplicate subsamples for cell enumeration (5 mL) were preserved immediately using Lugol’s Iodine and stored in the dark.

Subsamples (1 mL) for direct freezing were frozen at −20 °C, −80 °C or in liquid nitrogen. Formic acid (FA; 1 µL) was added and microcystins were extracted by three freeze-thaw cycles interspersed with a sonication step (30 min; Transonic T 700/H ultrasonic bath; Elma, Wetzikon, Switzerland). The samples were centrifuged (6000× *g*, 5 min; Eppendorf Minispin Plus, Hamburg, Germany) and the supernatant (800 µL) transferred to a septum-capped vial containing methanol (MeOH; 800 µL).

Subsamples (1 mL) processed by centrifugation were spun at high speed to pellet the cyanobacterial biomass (6000× *g*, 5 min). The supernatant was removed before cell pellets were frozen in liquid nitrogen and lyophilized (FreeZone6 freeze-drier; Labconco, Kansas City, MO, USA). The lyophilized sample was sonicated in MeOH (1 mL; 30 min). After extraction, samples were centrifuged (6000× *g*, 5 min) and 800 µL of supernatant placed in a septum-capped vial containing deionized water (800 µL). 

Subsamples (1 mL) processed by filtration were syringe filtered onto glass fiber filter paper (GF/C, 25 mm diameter; MicroScience, Mississauga, Canada). The filter was placed in an Eppendorf tube (1.5 mL), frozen in liquid nitrogen and lyophilized. The lyophilized sample was sonicated in MeOH (1 mL; 30 min). After extraction, samples were centrifuged (6000× *g*, 5 min) and 800 µL of supernatant was placed in a septum-capped vial containing deionized water (800 µL). The filtrate from these samples was syringe filtered (0.2-µm) and diluted in MeOH (1:1) in a septum-capped vial. The microcystin concentration detected in the filtrate was subtracted from the amount detected in directly frozen samples to adjust for extracellular microcystins in the culturing medium and to allow determination of the cellular microcystin quota.

Control samples (1 mL) were frozen in liquid nitrogen, lyophilized and extracted in MeOH (1 mL) with sonication (30 min). Samples were centrifuged (6000× *g*, 5 min) and the supernatant (800 µL) was placed in a septum-capped vial containing deionized water (800 µL). The control was used to ensure that the two extraction procedures (aqueous and organic) yielded similar extraction efficiencies and to normalize the microcystin results obtained from different strains/days.

### 3.3. Microcystin Adherence to GF/C Filters

An extract containing hydrophilic and hydrophobic microcystins was prepared by centrifuging a sample of *Microcystis* CAWBG11 (15 mL; 3200× *g*, 10 min). The cell pellet was vortexed in 100% MeOH (15 mL) and sonicated for 30 min, before the extract was clarified by centrifugation (3200× *g*, 10 min). The extract was separated into two aliquots (9.99 mL each) and supplemented with either 10 µL deionized water or 10 µL of formic acid (final concentration of 0.1%). Triplicate subsamples of each extract (1 mL each) were sonicated (30 min) with or without a GF/C filter. After removal of the GF/C filter, the samples were centrifuged (6000× *g*, 5 min) and the supernatant (800 µL) was placed in a septum-capped vial containing deionized water (800 µL) for microcystin analysis.

### 3.4. Temporal Effect of Centrifugation

Fifteen subsamples (1 mL; triplicate samples for five time-points) of a batch culture of *Microcystis* CAWBG16 were centrifuged (6000× *g*, 5 min). The cell pellets were left at ambient temperature covered with supernatant for 0, 1, 2, 3, 4 and 5 h, before the supernatant was removed and the cell pellet was frozen in liquid nitrogen. The sample was lyophilized and sonicated in MeOH (1 mL; 30 min). After extraction, samples were centrifuged (6000× *g*, 5 min) and the supernatant (800 µL) was placed in a septum-capped vial containing deionized water (800 µL) for microcystin analysis.

On a separate day the experiment was repeated, except that the supernatant was removed from all samples after centrifugation, but was not washed. The exposed cell pellets were incubated at ambient temperature for 0, 1, 2, 3, 4 and 5 h, before the cell pellet was frozen in liquid nitrogen and lyophilized. The lyophilized sample was extracted by sonication in MeOH (1 mL; 30 min). After extraction, samples were centrifuged (6000× *g*, 5 min) and the supernatant (800 µL) was placed in a septum-capped vial containing deionized water (800 µL).

### 3.5. Microcystin Analysis

Microcystin analysis was performed on an AmaZon X electrospray ionization mass spectrometer (Bruker, Ballerica, MA, USA) coupled to an UltiMate 3000 HPLC system (Dionex, Sunnyvale, CA, USA). Samples (20 µL) for LC-MS/MS were separated on a C_18_ column (Aeris PEPTIDE XB-C18, 100 × 2.1 mm, 3.6-μm; Phenomenex, Torrance, CA, USA) at a flow rate of 250 µL/min and a column temperature of 40 °C. A gradient of 2% acetonitrile +0.1% FA (solvent A) and 98% acetonitrile +0.1% FA (solvent B) operated with the following program; the sample was loaded in 20% B; held for 1 min; increased to 30% B over 1 min; increased to 60% B over the following 10 min; after increasing to 100% B over 1 min this solvent concentration was maintained for 2 min before the composition was returned to 20% B in 1 min and the column re-equilibrated for 4 min (20 min total). The eluting compounds were ionized using a capillary voltage of 3.5 kV and a nebulizer pressure of 3 bar. Desolvation was accomplished with a nitrogen flow of 8 L/min at 220 °C.

Microcystins were quantified using a multiple reaction monitoring (MRM) method which assessed the [M − H_2_O − H]^−^ ions of 21 microcystin congeners present in the cyanobacterial strains assessed. To enable direct comparison among strains the sum of the concentration of the different congeners (*i.e.*, total microcystins) was utilized. The microcystins assessed by LC‑MS/MS and their respective MRM transitions were: MC‑RR 1036.5 > 1018.5; dmMC‑RR 1022.5 > 1004.5; didmMC‑RR 1008.5 > 990.5; MC‑LR 993.5 > 975.5; dmMC‑LR 979.5 > 961.5; didmMC‑LR 965.5 > 947.5; MC‑YR 1043.5 > 1025.5; MC‑FR 1027.5 > 1009.5; MC‑WR 1066.5 > 1048.5; MC-RA 951.5 > 933.5; MC‑RAba 965.5 > 947.5; MC‑LA 908.5 > 890.5; dmMC‑LA 894.5 > 876.5; MC‑YA 958.5 > 940.5; MC‑FA 942.5 > 924.5; MC‑WA 981.5 > 963.5; MC‑LAba 921.5 > 903.5; MC‑FAba 956.5 > 938.5; MC‑WAba 995.5 > 977.5; MC‑FL 984.5 > 966.5; MC‑WL 1023.5 > 1005.5.

Standard curves were constructed by analyzing a mixture of three microcystin standards (MC‑LR, MC‑RR and MC‑LA; DHI Lab Products, Hoersholm, Denmark). The MC-RR standard was used to quantify MC‑RR, dmMC‑RR and didmMC‑RR. The MC-LR standard was used to quantify MC‑LR, dmMC‑LR, didmMC‑LR, MC‑YR, MC‑FR, MC‑WR, MC‑RA and MC‑RAba. The MC‑LA standard was used to quantify MC‑LA, dmMC‑LA, MC‑YA, MC‑FA, MC‑WA, MC‑LAba, MC‑FAba, MC‑WAba, MC‑FL and MC‑WL.

### 3.6. Cell Enumeration 

Subsamples (1 mL) of CAWBG11, a colonial *Microcystis*, were mechanically ground for 30 s using a teflon/glass tissue grinder (Wheaton, Rochdale, UK) to assist in cell enumeration. Subsamples (0.1–1 mL) from all strains were settled in plankton chambers (Hydrobios GmbH, Kiel, Germany). For CAWBG11 and CAWBG16, cells from ten random fields of view were counted at 400× magnification (IX71 inverted microscope; Olympus, Tokyo, Japan). For CAWBG59, trichome lengths were measured and divided by the average cell length (measured at 1000× magnification on 30 filaments using an BX51 light microscope; Olympus, Tokyo, Japan) to give an estimate of the number of cells per filament. The total number of cells was calculated by multiplying the number of cells per filament by the number of filaments.

### 3.7. Statistical Analyses

Statistical analyses were undertaken in R 2.12.1 [36]. To account for variation between experimental days and to enable pooling of all data points across strains, microcystin concentrations for each strain were normalized by dividing each value by that of the corresponding control sample (frozen in liquid nitrogen, lyophilized and methanol extracted). Analysis of variance was utilized to compare the median concentrations for each extraction treatment. Post-hoc pairwise comparisons were undertaken using Tukey honest significant difference.

## 4. Conclusions 

All preservation/enrichment methods tested, except for GF/C filtration, resulted in comparable microcystin quotas for each cyanobacterial strain tested. This shows that cyanobacterial biomass enrichment, as part of a sample preparation procedure for microcystin analysis, does not alter microcystin metabolism and that the microcystin measurements are still reliable. However, we identified that arginine-containing microcystins adhere to GF/C filters. Therefore, researchers should take caution when using GF/C filtration and ensure that careful method validation is conducted. The most widely applicable sampling method tested was direct freezing, as this can be utilized in both field and laboratory environments. Biomass enrichment by centrifugation did not stimulate microcystin metabolism over the normal sample processing time-frame. However, when the supernatant from centrifugation samples was removed quick preservation is recommended, as an increase in microcystin concentration was observed within 2 h.

## References

[1] Chorus I., Bartram J. (1999). Toxic Cyanobacteria in Water: A Guide to Their Public Health Consequences, Monitoring and Management.

[2] Falconer I.R., Yeung D.S.K. (1992). Cytoskeletal changes in hepatocytes induced by *Microcystis* toxins and their relation to hyperphosphorylation of cell proteins. Chem. Biol. Interact..

[3] Yoshizawa S., Matsushima R., Watanabe M.F., Harada K.-I., Ichihara A., Carmichael W.W., Fujiki H. (1990). Inhibition of protein phosphatases by microcystis and nodularin associated with hepatotoxicity. J. Cancer Res. Clin. Oncol..

[4] Ueno Y., Nagata S., Tsutsumi T., Hasegawa A., Watanabe M.F., Park H.-D., Chen G.-C., Chen G., Yu S.-Z. (1996). Detection of microcystins, a blue-green algal hepatotoxin, in drinking water sampled in Haimen and Fusui, endemic areas of primary liver cancer in China, by highly sensitive immunoassay. Carcinogenesis.

[5] Falconer I.R. (1999). An overview of problems caused by toxic blue-green algae (cyanobacteria) in drinking and recreational water. Environ. Toxicol..

[6] Grosse Y., Baan R., Straif K., Secretan B., El Ghissassi F., Cogliano V. (2006). Carcinogenicity of nitrate, nitrite, and cyanobacterial peptide toxins. Lancet Oncol..

[7] Msagati T.A.M., Siame B.A., Shushu D.D. (2006). Evaluation of methods for the isolation, detection and quantification of cyanobacterial hepatotoxins. Aquat. Toxicol..

[8] Spoof L., Meriluoto J., Codd G.A. (2005). Microcystins and nodularins. TOXIC: Cyanobacterial Monitoring and Cyanotoxin Analysis.

[9] Sangolkar L.N., Maske S.S., Chakrabarti T. (2006). Methods for determining microcystins (peptide hepatotoxins) and microcystin-producing cyanobacteria. Water Res..

[10] Moollan R.W., Rae B., Verbeek A. (1996). Some comments on the determination of microcystin toxins in waters by high-performance liquid chromatography. Analyst.

[11] Ortea P.M., Allis O., Healy B.M., Lehane M., Ní Shuilleabháin A., Furey A., James K.J. (2004). Determination of toxic cyclic heptapeptides by liquid chromatography with detection using ultra-violet, protein phosphatase assay and tandem mass spectrometry. Chemosphere.

[12] Rapala J., Erkomaa K., Kukkonen J., Sivonen K., Lahti K. (2002). Detection of microcystins with protein phosphatase inhibition assay, high-performance liquid chromatography-UV detection and enzyme-linked immunosorbent assay: Comparison of methods. Anal. Chim. Acta.

[13] Spoof L., Vesterkvist P., Lindholm T., Meriluoto J. (2003). Screening for cyanobacterial hepatotoxins, microcystins and nodularin in environmental water samples by reversed-phase liquid chromatography-electrospray ionisation mass spectrometry. J. Chromatogr. A.

[14] Metcalf J.S., Codd G.A. (2000). Microwave oven and boiling waterbath extraction of hepatotoxins from cyanobacterial cells. FEMS Microbiol. Lett..

[15] Barco M., Lawton L.A., Rivera J., Caixach J. (2005). Optimization of intracellular microcystin extraction for their subsequent analysis by high-performance liquid chromatography. J. Chromatogr. A.

[16] Qu J., Zhang Q., Jia C., Liu P., Yang M. (2013). Optimization of microcystin extraction for their subsequent analysis by HPLC-MS/MS method in urban lake water. Int. J. Environ. Sci. Dev..

[17] Li F., Liu W., Zhao N., Duan J., Wang Z., Zhang Y., Xiao X., Liu J., Yin G., Shi C. (2013). Studies on extracting microcystin-LR From *Microcystis aeruginosa* by water bath. J. Environ. Prot..

[18] Kim I.S., Nguyen G.H., Kim S.Y., Lee J., Yu H.W. (2009). Evaluation of methods for cyanobacterial cell lysis and toxin (microcystin-LR) extraction using chromatographic and mass spectrometric analyses. Environ. Eng. Res..

[19] Wood S.A., Dietrich D.R., Cary S.C., Hamilton D.P. (2012). Increasing *Microcystis* cell density enhances microcystin synthesis: A mesocosm study. Inland Waters.

[20] Wood S.A., Rueckert A., Hamilton D.P., Cary S.C., Dietrich D.R. (2011). Switching toxin production on and off: Intermittent microcystin synthesis in a *Microcystis* bloom. Environ. Microbiol. Rep..

[21] Pereira D.A., Giani A. (2014). Cell density-dependent oligopeptide production in cyanobacterial strains. FEMS Microbiol. Ecol..

[22] Puddick J., Prinsep M.R., Wood S.A., Kaufononga S.A.F., Cary S.C., Hamilton D.P. (2014). High levels of structural diversity observed in microcystins from *Microcystis* CAWBG11 and characterization of six new microcystin congeners. Mar. Drugs.

[23] Wood S.A., Rhodes L.L., Adams S.L., Adamson J.E., Smith K.F., Smith J.F., Tervit H.R., Cary S.C. (2008). Maintenance of cyanotoxin production by cryopreserved cyanobacteria in the New Zealand culture collection. N. Z. J. Mar. Freshwat. Res..

[24] Wood S.A., Heath M.W., Holland P.T., Munday R., McGregor G.B., Ryan K.G. (2010). Identification of a benthic microcystin-producing filamentous cyanobacterium (*Oscillatoriales*) associated with a dog poisoning in New Zealand. Toxicon.

[25] Shi L., Carmichael W.W., Miller I. (1995). Immuno-gold localization of hepatotoxins in cyanobacterial cells. Arch. Microbiol..

[26] Young F.M., Thomson C., Metcalf J.S., Lucocq J.M., Codd G.A. (2005). Immunogold localisation of microcystins in cryosectioned cells of *Microcystis*. J. Struct. Biol..

[27] Zilliges Y., Kehr J.-C., Meissner S., Ishida K., Mikkat S., Hagemann M., Kaplan A., Börner T., Dittmann E. (2011). The cyanobacterial hepatotoxin microcystin binds to proteins and increases the fitness of *Microcystis* under oxidative stress conditions. PLoS One.

[28] Walsby A.E. (1994). Gas vesicles. Microbiol. Rev..

[29] Wood S.A. Personal observation on the difficulty in centrifuging buoyant colonial *Microcystis*.

[30] Walsby A.E. (1991). The mechanical properties of the *Microcystis* gas vesicle. J. Gen. Microbiol..

[31] Harada K.-I., Kondo F., Lawton L., Chorus I., Bartram J. (1999). Laboratory analysis of cyanotoxins. Toxic Cyanobacteria in Water: A Guide to Their Public Health Consequences, Monitoring And Management.

[32] Hyenstrand P., Metcalf J.S., Beattie K.A., Codd G.A. (2001). Effects of adsorption to plastics and solvent conditions in the analysis of the cyanobacterial toxin microcystin-LR by high performance liquid chromatography. Water Res..

[33] Heussner A.H., Altaner S., Kamp L., Rubio F., Dietrich D.R. (2014). Pitfalls in microcystin extraction and recovery from human blood serum. Chem. Biol. Interact..

[34] Hyenstrand P., Metcalf J.S., Beattie K.A., Codd G.A. (2001). Losses of the cyanobacterial toxin microcystin-LR from aqueous solution by adsorption during laboratory manipulations. Toxicon.

[35] Bolch C., Blackburn S. (1996). Isolation and purification of Australian isolates of the toxic cyanobacterium; *Microcystis aeruginosa*; Kütz. J. Appl. Phycol..

[36] Institute for Statistics and Mathematics of Wirtschaftsuniversität Wien The R Project for Statistical Computing. http://www.r-project.org/.

